# Pruning and dormancy breaking make two sustainable grape-cropping productions in a protected environment possible without overlap in a single year

**DOI:** 10.7717/peerj.7412

**Published:** 2019-08-02

**Authors:** Zhipeng Qiu, Guangzheng Chen, Dongliang Qiu

**Affiliations:** 1 College of Horticulture, Fujian Agriculture and Forestry University, Fuzhou, Fujian, China; 2 Xiamen Lunong Agricultural Science and Technology Co.Ltd, Xiamen, Fujian, China; 3 Jiu’an Company of Taiwan, Nantou, Taiwan, China

**Keywords:** Two cropping, Summer black (*Vitis vinifera L*.), Flower sprouting, Degreening, Number of buds retained at pruning, Dormancy breaking, Maturation regulation, Fruit quality

## Abstract

In table grape production, protected cultivation in a vineyard in different regions and climates is currently a commonly used practice. The aims of this study were to provide key approaches to sustainably produce two crops of grape without overlap under protected environment in a single year. Spraying the degreening chemicals 400 mg/L ethephon +0.4% sulfur at 4 weeks of vine nutrient restoration after the harvest of the summer crop resulted in the highest percentage of sprouting inflorescence. The retention of 7–10 buds in the base shoot results in the high percentage of sprouting inflorescence. Bud breaking chemicals with 2.5% hydrogen cyanamide+2.0% Baoguoliang +0.02% Shenzhonggen significantly led to sprout inflorescence more efficiently. Cluster and fruit weights of the winter crop weighed significantly less than those of the summer crop. However, the contents of total soluble sugar and titratable acidity were higher than those of the summer crop. The anthocyanin content in the peel of the winter fruit was significantly higher than that in the summer fruit. The yield of the winter crop is controlled by the yield of the summer fruit. To maintain the stability of the two crops for one year, the ratio of yield in the winter to the summer should be controlled from 2:5 to 3:5 to ensure the sustainable production of two crops without overlap for ‘Summer Black’ grape. These results may help grape growers to overcome the impacts of rainy and hot climates with the help of protected facilities, and it could enable the use of solar radiation and heat resources in subtropical and tropical areas.

## Introduction

Grape is one of the popular fruit crops grown around the world due to its economic importance and the favorable effects on human health.

Table grapes are widely produced and consumed in China, which is the largest country that produces table grapes with 9.2 million tons worldwide, accounting for 34% in 2014. Its production in China occupied 80% of total grape production. By the end of 2016, the viticulture area in China reached 552,000 ha, increasing by 20,000 ha per year. In recent years, Yunnan, Guangxi, Hunan, Zhejiang, Fujian and other southern producing areas have had a strong momentum of development, with a total area of approximately 36.6% of the country, and 38.4% of the country’s fresh table grape production.

In table grape production, protected cultivation in a vineyard in different regions and climates is currently a commonly used practice with higher water use efficiency and better berry quality because the plastic film covers protect the vines and fruits from adverse conditions like wind, rain, hail, frost, scorching sunlight, pest and diseases (Du et al., 2015; Genta et al., 2010; Novello & De Palma, 2008; Roberto, Colombo & De Assis, 2011). This method effectively overcomes the problems of rain, and produces satisfactory economic and social benefits (Sun et al., 2006; Meng et al., 2013).

**Figure 1:**
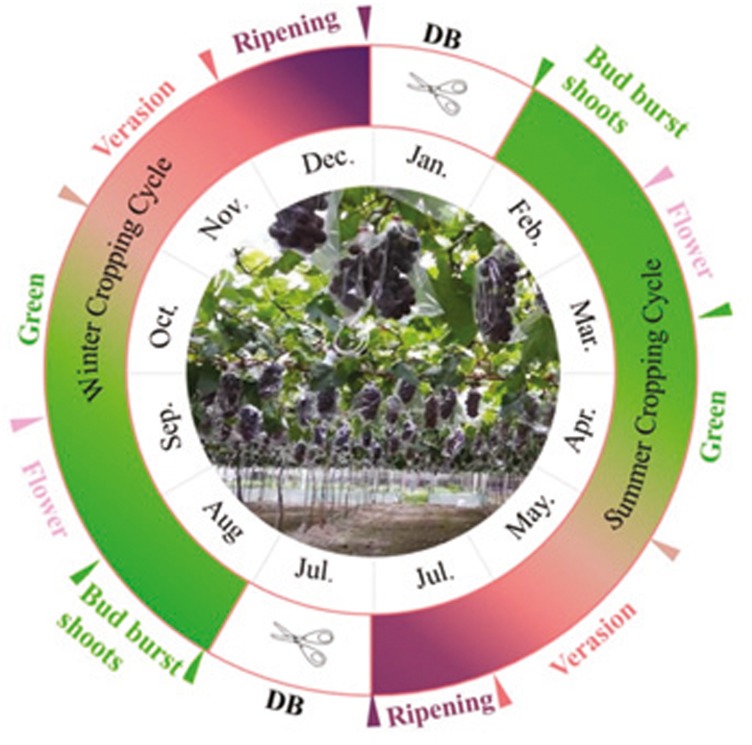
The schematic diagram of two cropping production of grapevine without overlap. Modified from Chen et al. (2017). DB: dormancy breaking.

The grapevine forms mixed bud with the characteristics of multiple differentiation. Some approaches were conducted to harvest twice a year not only for table grape production but also for good wine production (Dunuyaali, Okamoto & Shimamura (1983); Lin et al., 1985; Lin, 1987; Favero et al., 2011; Bai et al., 2008; Yan et al., 2014). Recently, for winemaking purposes, the winter fruits in Brazilian showed physicochemical traits more favorable than those from the summer season, because the summer fruits had higher cluster weight and titratable acidity while the winter ones featured a higher total soluble solids (TSS) content and pH value (Junior et al., 2017; Mitra et al., 2018). However, the approaches could not sustainably produce two crops in the following year. In addition, due to the overlap in fruit in the grapevine (some fruits are maturing while some are still in the fruit-setting stage), it is difficult for the grapevine to provide the nutrients for the development and ripening of fruit. The more berry fruits born in the first season, the fewer yields are obtained in the second harvest. The fruits in the second harvest could not ripen at the same time. In addition, disease and pest control, and other cultural practices are difficult to manage. Thus, the fruit quality from both seasons is poor, though an additional harvest is obtained. An alternative to overcome these problems involves the integrative use of the practice of pruning, defoliation and dormancy-breaking chemicals that induce flower formation after the first crop (Fig. 1).

Pruning is one of the most important practices of vine management to obtain consistently good yields and fruit quality in each growing season. The dormant pruning is conducted before the growth begins. As one of the cultural operations conducted in the vineyards, training and pruning has important implication for vine function since it influences the form and size of the vine, the balance between vegetative and fruit growth in the vine, and the quantity and quality of grape production. The ratio between the vegetative organs and crop weight has crucial importance for grape quality, and the ratio can be affected through the retention of different numbers of buds retained at pruning, the variation in shoot numbers, defoliation, or by affecting the cluster number per vine (Silvestroni et al., 2016; Silvestroni, Lanari & Pallioti, 2018; Frioni et al., 2016; Palliotti et al., 2017; Rahmani, Bakhshi & Qolov, 2015; Tassie & Freeman, 2001).

In many areas, various pruning systems are used for table grape cultivars, including 2–3 buds, half cane (6–8 buds) and cane (14–16 buds) systems, depending on the cultivar and region, since fruitful cultivars are spur-pruned while less fruitful cultivars are half cane or cane pruned. However, few comparative studies have been conducted that examine the changes in the flower sprouting due to grape shoot responses to different pruning severities. In tropical countries, grapevines are evergreen and they are pruned while they are actively growing. Defoliation is a highly effectively tool to dominate the vegetative stage. In such summer pruning, the defoliation of the mature leaves and the application of fertilizers are recommended to promote bud bursting (Dunuyaali, Okamoto & Shimamura, 1983).

Among the plant growth regulators that stimulate and standardize the budding and flowering of grape, hydrogen cyanamide (HC) is one of the most widely used due to its high efficiency of breaking dormancy. Other alternatives to HC include nitrogen compounds (calcium cynamide), mineral oil, and garlic extract (Leonei, Tecchio & Coser, 2015; Mohamed, 2008). Within this context, this study evaluates the influence of applying different doses of foliar fertilizer (Baoguoliang, BGL) and HC on the breaking dormancy, phenology and the yields of grapes grown in protected facilities.

Therefore, this study aimed to evaluate the effects of degreening chemicals, vine nutrient restoration time, different pruning levels, and dormancy breaking chemical on the initiation of sterile buds, and the optimization of cultivation techniques on the ‘one year two crops without overlap in a calendar year’ method of grape cultivation under protected facilities and to improve the quality of grapes and regulate the period of production.

## Materials and Methods

### Field location

**Figure 2:**
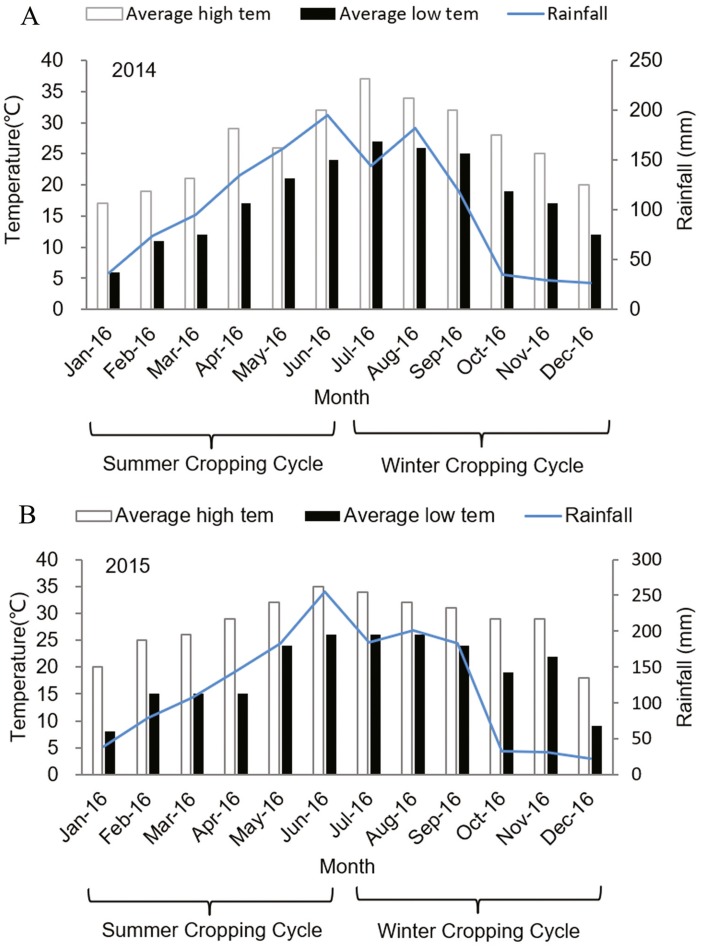
Monthly air temperature and rainfall of experimental site in (A) 2014 and (B) 2015.

The experiment was conducted at the Haidi Grape Orchard, situated at 24°79′09″N, 118°24′25″E and at 58 m altitude, Xiamen, Fujian Province between 2013 and 2016. Field study was approved by the authority of Haidi Grape Orchard (approval #01). The climate in this region is subtropical oceanic monsoon climate, which indicates that it is humid subtropical, dry in the winter and rainy in the summer. The average annual rainfall is 1,440 mm. The relative humidity is 78% and the average temperature in the coldest month (January) is 9.9 °C, and the hottest month (July) is 32.3 °C, while the average annual temperature is 21 °C (Fig. 2). The soil of the area is classified as red-yellow one.

**Figure 3:**
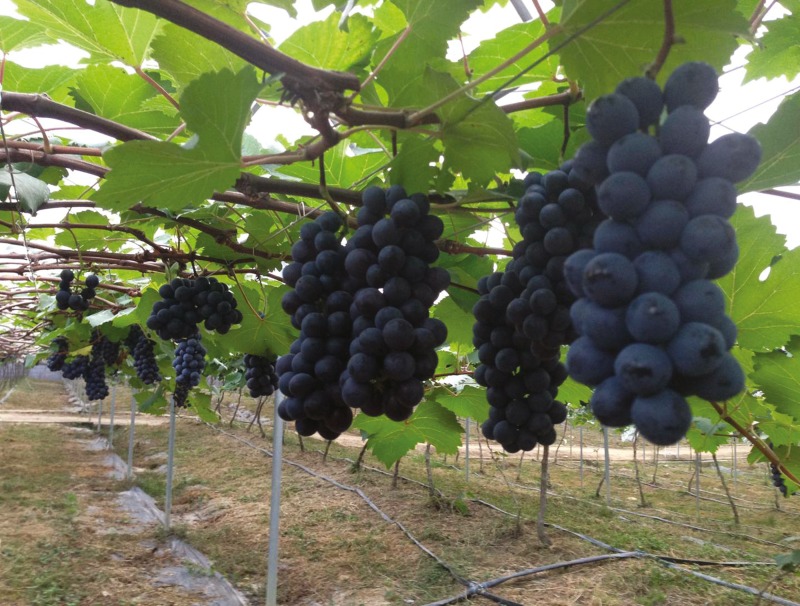
Vines growing with T-type horizontal trellises in Haiti vineyard, Xiamen, Fujian.

The vines of ‘Summer Black’ (*Vitis vinifera* L.) were grown with 2.0 m spacing within rows and 3.0 m spacing between rows in two multi-span steel sheds with a 120-mm thick, low-density polyethylene-vinyl house structure. The length and width of one multi-span steel shed were 105 m and 115 m, and the values for the other shed were 85 m and 141 m. A single arch shed was designed to be consistent with the trellises of the grapevines with 6.0 in width, 3.8 m in height above the ground and 2.4 m in the height of its shoulder. This structure could block rainfall and provide a moderately humid environment in the rainy season, and keep the plants warm in the winter. The air movement in the greenhouse could be regulated by artificially opening the top and side windows. A T-type, horizontal trellis with 1.9 m in height was adopted in this vineyard (Fig. 3). All rows of the vineyard were oriented from north to south in both sheds. There were three replicates of each treatment arranged in a randomized block design. Each replicate was a panel of nine vines with similar growth vigor. A total of 27 vines were selected for data collection in each treatment. The vineyard was managed using standard viticulture practices for the cultivar and region. Pest management was conducted using local standard practices.

### The de-greening chemical trials

The de-greening chemicals 400 mg/L ethephon, 0.4% sulfur and their combination of 400 mg/L ethephon and 0.4% sulfur were applied 7, 6, 5, 4, 3, 2, 1 and 0 weeks after the harvest of summer crop. At each application, leaves were thoroughly wetted. All leaves developed on the main shoot on the vine that was sprayed with degreening chemicals were removed at the time of pruning.

### Pruning

The severity of pruning was intended as the number of buds retained on the base shoot at pruning. Pruning for the summer crop was conducted in January, one month before the shoots sprouted. Seven treatments were used with 2, 3, 4, 5, 6, 7, 8 retained on the base shoot. Pruning for the winter crop was conducted one week before the shoot sprouted, and 6 treatments were used with 5, 6, 7, 8, 9, 10 buds retained on the base shoot.

### Dormancy breaking

At the same day of pruning, the top bud of cane was dotted with different dormancy breaking chemicals to promote the breaking of dormancy, and those buds were marked by carmine. Seven treatments consisted of 2.5% hydrogen cynamide (HC), 2.0% Baoguoliang (BGL) with its active ingredient of water soluble phosphoric acid anhydride, 2.5% HC+2.0% BGL, 2.5% HC + 0.02% Shengzhonggen (SZG, an interfacial activator), 2.0% BGL + 0.02% SZG, 2.5% HC+2.0% BGL+0.02% SZG, and water as a control.

### Yield of the summer crop affecting the flower sprouting of the winter crop

To investigate the effect of the yield of the summer crop on sprouting flower cluster of the winter crop, 10, 15, 20, 25, 30, 35 and 40 berry clusters per vine of the summer crop were retained, respectively, and all the other clusters on the same vine were removed. The clusters were manually removed at the**** pea-size**** stage (approximately 4 weeks after flowering).

### Observations

The phenological stages were identified on the basis of Lorenz et al. (1995) and Vasconcelos et al. (2009), and shown in Table 1. The buds were counted on each base shoot of the vine that was previously marked. The percentages of sterile bud break (PSBB), fertile bud break (PFBB) and sprouting inflorescence (PSI) were obtained using the following formula:

**Table 1:** Key grapevine growth stages from the BBCH scale and additional growth stages.

BBCH grapevine growth stage****		Alternative interpretation of modified E-l growth stages for reference in this study****	
BBCH code****	Description	BBCH code	Designation
05****	Brown wool clearly visible	05	Budding onset
15****	Five leaves unfold	15	Full budding
60****	First flowerhoods detached from the receptacle	60	Full bloom
73****	Berry groat-sized	73	Onset of berry setting
75****	Berry pea-sized	75	Peak at berry setting
81****	Beginning of ripening	81	Onset of veraison
89****	Berry ripen for harvest	89	Berry ripen for harvest

PSBB = NSB/NTBD ×100, PFBB = NFB/NTBD ×100, PSI = NI/ NTBD ×100, where NSB, NFB, NI and NTBD mean number of sterile bud, number of fertile bud, number of inflorescence and number of top bud dotted with dormancy breaking chemicals, respectively.

### Grapevine yield and yield components

The grape yield and its components were measured once at harvest time. The yield and cluster number per vine were measured on 9–30 vines. On each of these vines, one cluster was selected at random to count the berry number per bunch and the weight of 100 berries was measured. The yield was determined by the kilos of grape bunches produced per plant and the stand of 1,650 plants per hectare.

### Grape quality analysis

Samples were removed from 27 clusters in nine vines that were representative of each plot when harvested to determine the physical and chemical parameters related to the ripening state of the grapes. The collected berry samples came from 80 representative berries, and were divided into two sections. The first 50 berries were used to measure the physical characters of the berries by recording the individual berry weight and fruit firmness with a portable hardness tester and then the berries were crushed into juice to determine the amount of total soluble solid (TSS) using a hand-held refractometer (WYT-15; Quanzhou Optical Instrument Factory, China). The result was expressed in °Brix. The total acidity was determined by titration with NaOH solution. Ten milliliters of the sample was combined with the indicator bromothymol blue and neutral red and titrated with a 0.1 mol/L NaOH solution. The amount of NaOH (mL) was then converted to tartaric acid (Mitra et al., 2018). Skin from the rest of the 30 berry was manually peeled, immediately frozen in liquid N_2_ and stored at −80 °C until further analysis of anthocyanin.

### Extraction and estimation of the total anthocyanin

Anthocyanin extractions were performed with a protocol similar to that described by Liang et al. (2011) with slight modifications. Briefly, the frozen berry skin samples were ground in liquid nitrogen with a mortar and pestle. A 0.5 g powdered sample was homogenized in 10 mL of 1% HCl-methanol and mixed. It was vortex for 1 min, incubated in a shaker at 250 rpm for 2 h at 4 °C in the dark and then centrifuged at 4 °C for 20 min at 13,200 g. The supernatant was collected and stored at −40 °C until the time of analysis. This extract was used to estimate total anthocyanin content. Its content was measured using a pH differential method similar to that described by Giusti & Wrolstad (2001)*.*

### Statistic analysis

The data were analyzed by variance (ANOVA) using SPSS (version 16.0). The significance of the differences was determined according to Duncan’s multiple range test (DMRT). *P* values < 0.05 are considered to be significant. The results are presented as the means ± SD. Correlation coefficient was calculated by SPSS.

## Results

### Degreening chemicals and spraying time

Degreening chemicals and spraying time significantly affected inflorescence sprouting in the summer. Spraying degreening chemicals 4 weeks after the harvest of the summer crop produced the highest percentage of sprouting inflorescence. The percentage of spraying 400 mg/L ethephon +0.4% sulfur was 195.2%,significantly higher than those of 400 mg/L ethephon (107.3%) and 0.4% sulfur (110.4%). The percentage of spraying 400 mg/L ethephon +0.4% sulfur 3 weeks after harvest was 144.5%, significantly higher than those of 400 mg/L ethephon (97.5%) and 0.4% sulfur (86.8%). In addition, a significant difference was found between spraying the degreening chemicals 3 and 5 weeks after the summer harvest (Table 2). Interestingly, sprouting inflorescence could even be obtained in the treatment of spraying degreening chemicals immediately after harvest.

### Pruning severity

**Figure 4:**
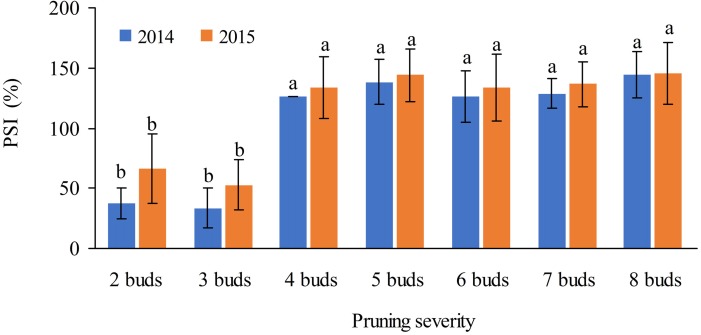
Effects of the number of buds retained at winter pruning on PSI for the summer crop of ‘Summer Black’ grape.

**Figure 5:**
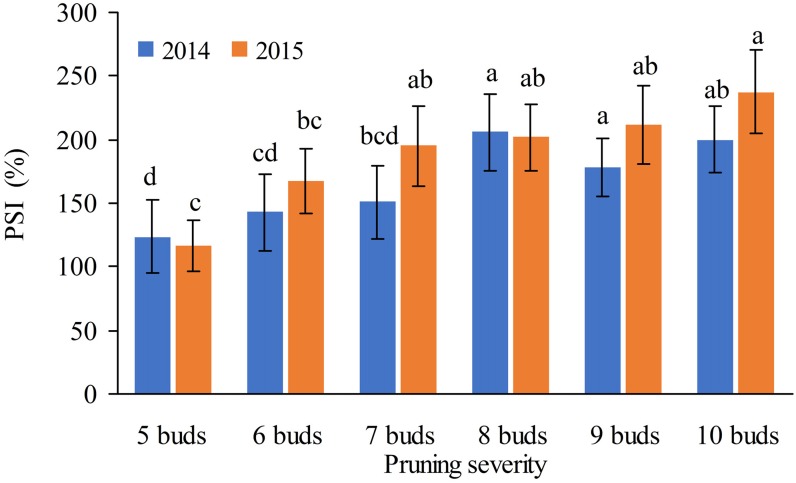
Effects of the number of buds retained at summer pruning on PSI for the winter crop.

The number of buds retained at pruning had a significantly effect on the inflorescence sprouting in both the summer and winter crops. In 2014 and 2015, retaining 4–8 of winter buds for the summer crop resulted in sprouting inflorescence with the percentage of more than 120.0%, significantly higher than that with 2–3 buds (Fig. 4). Retaining 7–10 summer buds for the winter crop resulted in sprouting inflorescence with a percentage of more than 150% in 2014 and 195% in 2015, significantly higher than that with 5–6 buds (Fig. 5).

**Table 2:** Effects of degreening chemicals and spraying time on the sprouting flowers of Summer Black grapes.

Degreening chemicals	Week after summer harvest	NTBD	NI	PSI
400 mg L^−1^ethephon	0	41	10	24.5 ± 0.7f
	1	38	23	60.5 ± 3.7de
	2	42	31	73.8 ± 10.1bc
	3	39	38	97.5 ± 3.5a
	4	42	45	107.3 ± 3.9a
	5	41	34	82.9 ± 4.0b
	6	42	28	66.6 ± 2.2dcd
	7	44	23	52.3 ± 3.2e
0.4% sulfur	0	42	11	26.2 ± 3.4d
	1	42	21	50.00 ± 0.0c
	2	42	32	76.4 ± 5.1b
	3	45	39	86.8 ± 5.9b
	4	39	43	110.4 ± 7.6a
	5	43	36	83.8 ± 2.8b
	6	42	33	78.6 ± 3.4b
	7	40	21	52.50 ± 3.5c
400 mg L^−1^ethephon + 0.4% sulfur	0	43	11	25.5 ± 2.4e
	1	42	19	45.00 ± 7.1de
	2	43	30	69.80 ± 2.3cde
	3	41	66	144.5 ± 50.1b
	4	42	82	195.2 ± 6.7a
	5	45	48	106.5 ± 9.2bc
	6	38	31	80.8 ± 20.0cd
	7	44	31	70.40 ± 0.6cde

**Notes.**

Values followed by different letters indicating significant difference between treatments (*p* < 0.05).

NTBD number of top bud dotted with dormancy breaking chemicals NI number of inflorescence PSI percentage of sprouting inflorescence

### Dormancy breaking

Dormancy breaking chemicals led to a significant increase in sprouting fertile and sterile buds in both the summer (Table 3) and winter (Table 4) crops. The use of 2.5% HC+2.0% BGL+0.02% SZG had the highest percentage of sprouting inflorescence in both the winter and summer crops. The percentage of the summer crop was 139.3% in 2013, 156% in 2014 and 177.3% in 2015 (Table 3), respectively, significantly higher than that of 2.5% HC, 2.0% BGL, 0.02% SZG individually, and the combination of both. The percentage of winter crop were 127.6% in 2013, 179.2% in 2014 and 190.5% in 2015, respectively, also significantly higher than that of 2.5% HC, 2.0% BGL, 0.02% SZG individually, and the combination of both (Table 4). As for sterile bud-breaking, the application of 2.5% HC+2.0% BGL+0.02% SZG, had the highest percentage of sprouting sterile bud in both the winter and summer crops However, no significantly differences were observed between the treatments of 2.5% HC+2.0% BGL+0.02% SZG ,2.5% HC+0.02% SZG and 2.5% HC in winter crop, and 2.5% HC+2.0% BGL+0.02% SZG, 2.5% HC+2.0% BGL and 2.5% HC+0.02% SZG in summer crop.****

**Table 3:** Effects of dormancy-breaking chemicals on the sprouting of the fertile and sterile buds of ‘Summer Black’ grape in the summer crop.

Treatments	PFBB (%)			PSBB (%)		
	2013	2014	2015	2013	2014	2015
Control	21.4 ± 9.6d	30.7 ± 13.8d	29.6 ± 13.0d	33.3 ± 14.6d	37.5 ± 8.0d	42.8 ± 8.3d
2.5% HC	58.6 ± 8.9c	79.3 ± 17.4c	119.0 ± 8.1b	157.1 ± 8.1b	171.4 ± 19.8b	185.7 ± 15.0b
2.0% BGL	73.3 ± 12.5c	75.0 ± 11.7c	76.5 ± 14.2c	78.9 ± 18.4c	89.5 ± 6.5c	91.3 ± 10.7c
2.5% HC+0.02% SZG	125.8 ± 8.6ab	134.5 ± 13.0ab	162.5 ± 16.6a	175.0 ± 17.5ab	225.0 ± 18.9a	237.5 ± 21.6a
2.0% BGL+0.02%SZG	80.6 ± 16.7c	83.3 ± 14.7c	89.3 ± 14.8c	85.7 ± 10.4c	86.7 ± 10.3c	107.7 ± 6.5c
2.5% HC+2.0% BGL	116.7 ± 14.4b	120.8 ± 19.4b	124.0 ± 21.8b	154.5 ± 4.0b	188.9 ± 8.7b	212.5 ± 16.5a
2.5% HC+2.0% BGL+0.02% SZG	139.3 ± 12.5a	156.0 ± 8.9a	177.3 ± 22.0a	187.5 ± 13.1a	237.5 ± 14.5a	212.5 ± 13.3a

**Notes.**

The data was investigated 35 days after application of dormancy-breaking chemicals. Values followed by different letters indicating significant difference between treatments (*p* < 0.05).

PFBB percentage of fertile bud break PSBB percentages of sterile bud break

**Table 4:** Effects of dormancy-breaking chemicals on the sprouting of the fertile and sterile buds of Summer Black grape in the winter crop.

Treatments	PFBB (%)			PSBB (%)		
	2013	2014	2015	2013	2014	2015
Control	30.9 ± 12.1d	38.2 ± 11.5d	40.0 ± 8.5e	36.3 ± 15.5e	40.5 ± 8.4f	43.8 ± 7.6e
2.5% HC	67.4 ± 18.7c	77.1 ± 6.1c	73.5 ± 17.8d	182.4 ± 16.0bc	198.7 ± 2.5b	205.6 ± 6.3b
2.0% BGL	78.4c ± 14.8c	83.3 ± 12.2c	115.3 ± 23.2c	127.9 ± 12.8d	109.3 ± 10.2e	111.3 ± 12.1d
2.5% HC+0.02% SZG	105.5 ± 5.4b	112.7 ± 11.2b	143.9 ± 5.0b	195.2 ± 5.4b	194.0 ± 6.1bc	207.1 ± 8.6b
2.0% BGL+0.02%SZG	82.3 ± 12.4c	89.7 ± 8.1c	93.4 ± 5.7d	128.7 ± 21.2d	166.5 ± 20.3d	172.6 ± 20.4c
2.5% HC+2.0% BGL	110.0 ± 3.6ab	118.8 ± 13.6b	148.3 ± 1.5b	159.5 ± 15.2c	173.4 ± 7.5cd	189.5 ± 10.3bc
2.5% HC+2.0% BGL+0.02% SZG	127.6 ± 4.4a	179.2 ± 16.2a	190.5 ± 9.4a	227.6 ± 20.6a	238.0 ± 20.4a	242.5 ± 13.9a

**Notes.**

The data was investigated 20 days after application of dormancy-breaking chemicals. Values followed by different letters indicating significant difference between treatments (*p* < 0.05).

PFBB percentage of fertile bud break PSBB percentages of sterile bud break

### The yield of the summer crop affects the flower sprouting of the winter crop

**Figure 6:**
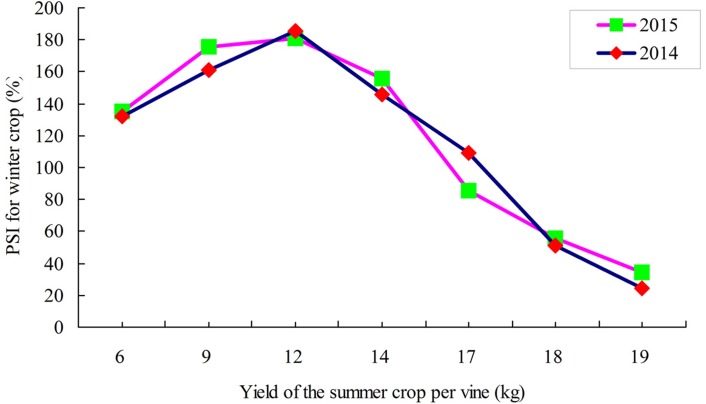
Correlation between the yield of the summer crop and PSI in the winter crop of ‘Summer Black’ grape.

The yield of the summer crop significantly affected the percentage of flower sprouting of the winter crop. The percentage of flower sprouting of the winter crop had a significantly negatively correlation with the yield of the summer crop (Table 5). The correlation coefficient in 2014 and 2015 was *r* =  − 0.799 (*p* < 0.05) and *r* =  − 0.778 (*p* < 0.05), respectively (Fig. 6).

**Table 5:** Effect of the yield of the summer crop on PSI in the winter crop of Summer Black grape in 2014 and 2015.

No of fruit cluster in the summer crop/vine	Yield of the summer crop per vine (kg)		PSI for winter crop (%)	
****	2014	2015	2014	2015
10	5.89 ± 1.63f	6.06 ± 2.40e	131.9 ± 18.9cd	135.10 ± 14.7c
15	8.64 ± 1.44de	8.89 ± 1.82de	161.1 ± 14.1ab	175.5 ± 9.5ab
20	10.96 ± 2.33de	11.70 ± 2.39cd	185.3 ± 16.6a	180.7 ± 11.3a
25	13.83 ± 1.93cd	14.08 ± 2.69bc	145.6 ± 13.6bc	155.8 ± 11.8bc
30	16.63 ± 0.71bc	16.61 ± 1.20ab	109.2 ± 22.0d	85.3 ± 20.2d
35	18.62 ± 1.58ab	18.35 ± 1.62a	51.3 ± 12.4e	55.6 ± 11.3e
40	20.77 ± 1.78a	19.03 ± 2.14a	24.6 ± 9.15e	34.2 ± 12.2e

**Notes.**

Values followed by different letters indicating significant difference between treatments (*p* < 0.05).

PSI percentage of sprouting inflorescence

### Comparison of the number of days from pruning to phenological stages between the two crops of Summer Black grape

For the summer crop, the vine started sprouting at 20 days in 2013, 18 days in 2014 and 20 days in 2015 after pruning. The budding, flowering, fruit setting, fruit colour breaking and harvesting peak of the plants treated with dormancy-breaking chemicals 2.5% HC+2.0% BGL+0.02% SZG occurred at 25, 54, 62, 98 , 124 days after pruning, respectively. The duration from flowering to the fruit ripening peak was 69 days. While for winter crop, the budburst began 8 days after pruning, which was 11 days earlier than that in the summer crop, and the budding, flowering, fruit setting, and fruit colour breaking in the winter crop peaked at 12, 31, 37, 61 days after pruning, and was 13, 23, 25, 37 days shorter than those in the summer crop, respectively. The duration from the peak of flowering to fruit ripening was 64 days in the winter crop, which was 5 days shorter than that in the summer crop (Table 6).

**Table 6:** Comparison in the number of days from pruning to the phenological stage between two crops treated with dormancy-breaking chemicals in a year.

Harvest season	Year	Budding onset	Full budding	Full flowering	Onset of berry setting	Peak at berry setting	Onset of veraison	Berry ripen for harvest	Duration from full flowering to ripen
Summer crop	2013	20	25	55	58	63	99	125	70
	2014	18	23	52	55	61	96	122	70
(First crop)	2015	20	26	54	56	62	98	124	68
Average		19	25	54	56	62	98	124	69
Winter crop	2013	7	12	30	33	36	60	93	63
	2014	8	12	31	34	37	61	95	64
(Second crop)	2015	8	11	32	35	38	61	96	64
Average		8	12	31	34	37	61	95	64

### Comparison of the yield component and fruit quality between the summer and winter crops

The berry cluster and berry weight of winter crop was 377 g and 5.8 g, significantly lower than those of the summer crop at 518 g and 7.4 g respectively. However, the content of the total soluble sugar of the winter fruit was 23.4%, significantly higher than that of the summer fruit, while the content of titratable acid was 8.9%, higher than that of the summer crop, showing no significant difference. The anthocyanin content in the peel of the winter fruit was 7.51 mg/g, significantly higher than that in the summer with 6.23 mg/g (Table 7).

**Table 7:** Comparison in the fruit quality and yield component between two crops in a year.

Year****	Harvest season****	Cluster weight(g)****	Berry weight (g)****	Fruit colour****	Fruit firmness (kg/cm^−2^)****	TSS (%)****	Titratable acid (mg/g)****	Anthocyanin content****(mg/g)****	Yield (Ton/ha)****
2014	Summer crop	521 ± 38a	6.9 ± 0.4a	Black	1.61 ± 0.06a	21.3 ± 0.5b	7.9 ± 0.7a	6.37 ± 0.14b	16.78 ± 2.71a
	Winter crop	373 ± 42b	5.3 ± 0.6b	Dark black	1.63 ± 0.09a	22.8 ± 0.3a	8.2 ± 0.6a	7.33 ± 0.20a	8.28 ± 1.65b
2015	Summer crop	518 ± 55a	7.4 ± 0.6a	Black	1.57 ± 0.06a	21.9 ± 0.2b	8.3 ± 0.7a	6.23 ± 0.15b	20.20 ± 2.60a
	Winter crop	377 ± 45b	5.8 ± 0.6b	Dark black	1.50 ± 0.08a	23.4 ± 1.1a	8.9 ± 0.3a	7.51 ± 0.18a	8.67 ± 1.81b

**Notes.**

Values followed by different letters indicating significant difference between treatments (*p* < 0.05).

**Figure 7:**
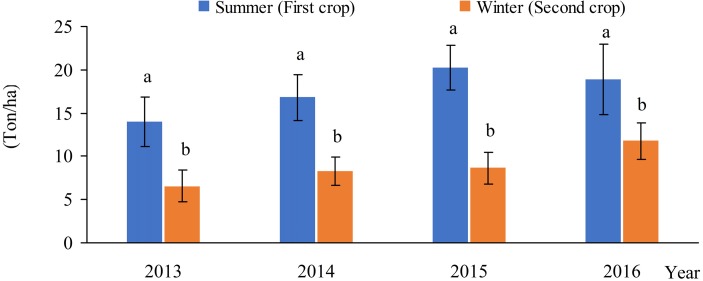
Comparison in the yield between two crops without overlap in a year.

The fruit ripening of the summer crop occurred from late May to July, while the fruit ripening of the winter crop occurred from November to January in the following year. The yield of the summer and winter crops of 2-age, 3-age, 4-age and 5-age of ‘Summer Black’ vine was 13.09 and 6.57 Ton/ha in 2013, 16.78 and 8.28 Ton/ha in 2014, 20.20 and 8.67 Ton/ha in 2015, 18.91 and 11.76 Ton/ha in 2016, respectively. The total yield in a calendar year went up accordingly with the increase in tree age (Fig. 6). The yield ratio of the winter to summer crops should be maintained from 2:5 to 3:5, and the total annual yield should be at 30 tons/ha of 5-age vine to ensure the sustainable production of two harvests without overlap for Summer Black grape (Fig. 7).

## Discussion

### Pruning plays a key role in sprouting inflorescence

Pruning is one of the key cultural practices that influence inflorescence sprouting, yield and fruit quality in grape production. In tropical and subtropical countries, removing old leaves at pruning was necessary to ensure good bud bursting for some fruit trees because the old grape leaves contained a high level of abscisic acid in the autumn, which is known to control the dormancy of the axillary bud (Dunuyaali, Okamoto & Shimamura, 1983). In this study, pruning severity impacted the sprouting inflorescence not only in the summer crop but also in the winter crop. Retaining 5–8 winter buds in base branch at pruning resulted in significantly higher percentage of sprouting inflorescence than that with 2–3 buds. While retaining 7–10 summer buds at pruning led to significantly higher percentage of sprouting inflorescences than that with 5-6 buds, demonstrating that the shoot with the summer buds sprouting inflorescence after pruning more than that with winter the buds.

### Chemical defoliation dominates vegetative growth, and enables the vine to re-sprout flower

The grapevine is a temperate deciduous fruit tree. It can achieve the effect of ‘winter’ by producing yellowing leaves in the summer. The fertile buds of grape in temperate areas are well differentiated. One of the primary factors is the gradual yellowing of the grape leaves with the autumn temperature and the process of the yellowing which transfers the nutrients in the leaves back to the buds and the branches. Therefore, leaf yellowing is an effective way to promote the fertile bud differentiation of grape by simulating the ‘winter’ process. After 3 weeks of restoration after harvest, the leaf was sprayed with degreening chemicals with 400 mg. ethephon + 0.4% sulfur. Simultaneously, irrigation in the soil was completely stopped until 5 days before summer pruning, prompting the grape leaves to undergo the ‘winter’ process for approximately 25 days. After a period of application of degreening chemicals, leaf yellowing occurred, the physiological function of plant declined, and large numbers of metabolic products accumulated. This process could then be used to induce flower initiation and to maximize yields. Therefore, the defoliation technique is an enormous tool for grape growers that enable the domination of the vegetative stage.

### Comparison of the roles of dormancy breaking chemicals between the summer and winter crops

It was reported that the presence of nitrogen compounds in products used to break dormancy, for example HC, caused respiratory stress, resulting in cell rearrangement and a ripple effect that could lead to breaking dormancy (Perez, Vergara & Or, 2009). In our experiments, before 5∼7 days of pruning, irrigation was performed until 15 days after bud sprouting for the second crop production. Dormancy-breaking chemicals were evenly smeared at the end of the branch on the same day as pruning. Without this application, uneven and reduced budburst levels were experienced. This result was supported by Burnett (1985), Mohamed et al. (2012), Smit (1985), Dry (1992) and Dokoozlian, Williams & Neja (1995). Among of them, the treatment of 2.5% HC+2.0% BGL+0.02% SZG was the most effective.

### Comparison in phenology between the summer crop and winter crops

The duration from the HC application to budburst and full flowering in the summer crop was longer than that in the winter crop. This was due to the lower temperatures in the stage of the flower development of the summer crop (February to April) as compared with that in the winter crop (August to October).The duration from full flowering (fruit set start) to the start of fruit veraison in the summer crop was also longer than that in the winter crop. However, the duration from veraison to ripening in the summer crop was shorter than that in the winter crop. This was due to lower temperatures in the stage of fruit enlargement of the summer crop (from April to May) than that of the winter crop (the end of September to October), and higher temperature in the stage of ripening (the middle of May to June) than that in the winter crop (November to December).

### Comparison on fruit quality between summer crop and winter crop

There was some difference on fruit characteristic between summer crop and winter crop. The berry weight from summer crop was significantly higher than those from winter crop, as well as the bunch weight. However, the TSS, and anthocyanin concentration from winter crop were significantly higher than those from summer crop. No difference of titratable acid between two crops was observed. This was resulted from the lower night temperature in the period of veraison in winter crop, which promoted accumulation of sugar and anthocyanin. Most of all, lower night temperature was detrimental to conversion of organic acid (Azuma, Yakushiji & Sato, 2019).

### The yield of the summer crop affects flower sprouting of the winter crop

Cluster number and berry number were the primary drivers of grape yield (Dry, 1992; Dokoozlian, Williams & Neja, 1995; Guilpart, Metay & Gary, 2014) and they were affected during the stages of the previous crop.**** Flower sprouting of the winter crop decreased significantly with the increase in cluster number of the summer crop. A significantly negative relationship was observed between the yield of summer crop and percentage of sprouting flowers in the following winter season in 2014 and 2015. This result indicated that the yield of the winter crop was controlled by the yield of the summer crop.**** To ensure that the two crops are sustainable, the ratio of fruit yield in the winter to that in the summer should be controlled from 2:5 to 3:5. Our results suggest that growers should not blindly increase the output of the summer fruit to avoid the sharp decrease in the fruit output in the winter. The two harvests without an overlap system in a calendar year could overcome the impact of rainy and hot climates with the help of protected facilities, and it could utilize the solar radiation and heat resource in subtropical and tropical areas.

## Conclusion

The new cultivation system of two crops a year required that there was no overlap in both flowers and fruits. The system could increase the yield and fruit quality, extend the supply of fresh fruits from the late spring to summer, autumn and winter, successfully regulate the table fruit market, extend the time for grape production for 4-5 months, stagger the peak of the market and promote the agricultural development of the facilities.

##  Supplemental Information

10.7717/peerj.7412/supp-1 Supplemental Information 1 Percentage of sprouting flower and fruit quality between two crops

## Additional Information and Declarations

### Field Study Permissions

The following information was supplied relating to field study approvals (i.e., approving body and any reference numbers): Field study was approved by the authority of Haidi Grape Orchard who manages the vineyard where the experiments were conducted (approval #01).

### Data Availability

The following information was supplied regarding data availability: The raw data are provided in a Supplemental File.
